# Evaluation of Structure-Function Relationships in Longitudinal Changes of Glaucoma using the Spectralis OCT Follow-Up Mode

**DOI:** 10.1038/s41598-018-35419-y

**Published:** 2018-11-21

**Authors:** Kenji Suda, Tadamichi Akagi, Hideo Nakanishi, Hisashi Noma, Hanako Ohashi Ikeda, Takanori Kameda, Tomoko Hasegawa, Akitaka Tsujikawa

**Affiliations:** 1 Department of Ophthalmology and Visual Sciences, Kyoto University Graduate School of Medicine, 54 Kawahara-cho, Shogoin, Sakyo-ku, Kyoto, 606-8507 Japan; 2 Department of Data Science, The Institute of Statistical Mathematics, 10-3 Midori-cho, Tachikawa, Tokyo, 190-8562 Japan

## Abstract

The detection of glaucoma progression is an essential part of glaucoma management. Subjectivity of standard automated perimetry (SAP) prevents the accurate evaluation of progression, thus the detection of structural changes by optical coherence tomography (OCT) is attracting attention. Despite its objectivity, there is controversy about the appropriateness of the use of OCT, because many previous studies have indicated OCT results may not reflect the deterioration of visual field. A reason for this dissociation may be the test-retest variability of OCT, a major cause of which is misplacement of the measurement location. Recent advantages of spectral-domain OCT (SD-OCT), especially Spectralis OCT with an eye-tracking system (follow-up mode) enable measurement at approximately the same location as previous examinations. In addition to utilizing Spectralis follow-up mode, we introduced structure-function relationship map and nonlinear relationship between SAP and OCT results in considering structure-function relationship in longitudinal changes. The introduction of these two ideas in our study population improved the correlation between the SAP and OCT (R = 0.589 at most). The results of this study support the practical use of OCT in glaucoma progression but also stress the importance of focus on the corresponding focal changes and the consideration of disease severity.

## Introduction

The detection of glaucoma progression is an essential part of glaucoma management, as the only evidence-based therapy for glaucoma is the prevention of progression by reduction of intraocular pressure (IOP)^1–3^. Standard automated perimetry (SAP) is most often used for the evaluation of glaucoma progression, but one of the disadvantages of SAP is its subjectivity^4,5^. Fluctuations of the examination results hamper the accurate evaluation of glaucoma progression^6,7^. A strategy focused on the detection of structural changes in the optic disc or retina by optical coherence tomography (OCT) is attracting attention^8,9^. Despite its objectivity, there is controversy about the appropriateness of the use of OCT to assess glaucoma progression, because many previous studies have reported disagreements between SAP and OCT results^10–13^.

**Figure 1:**
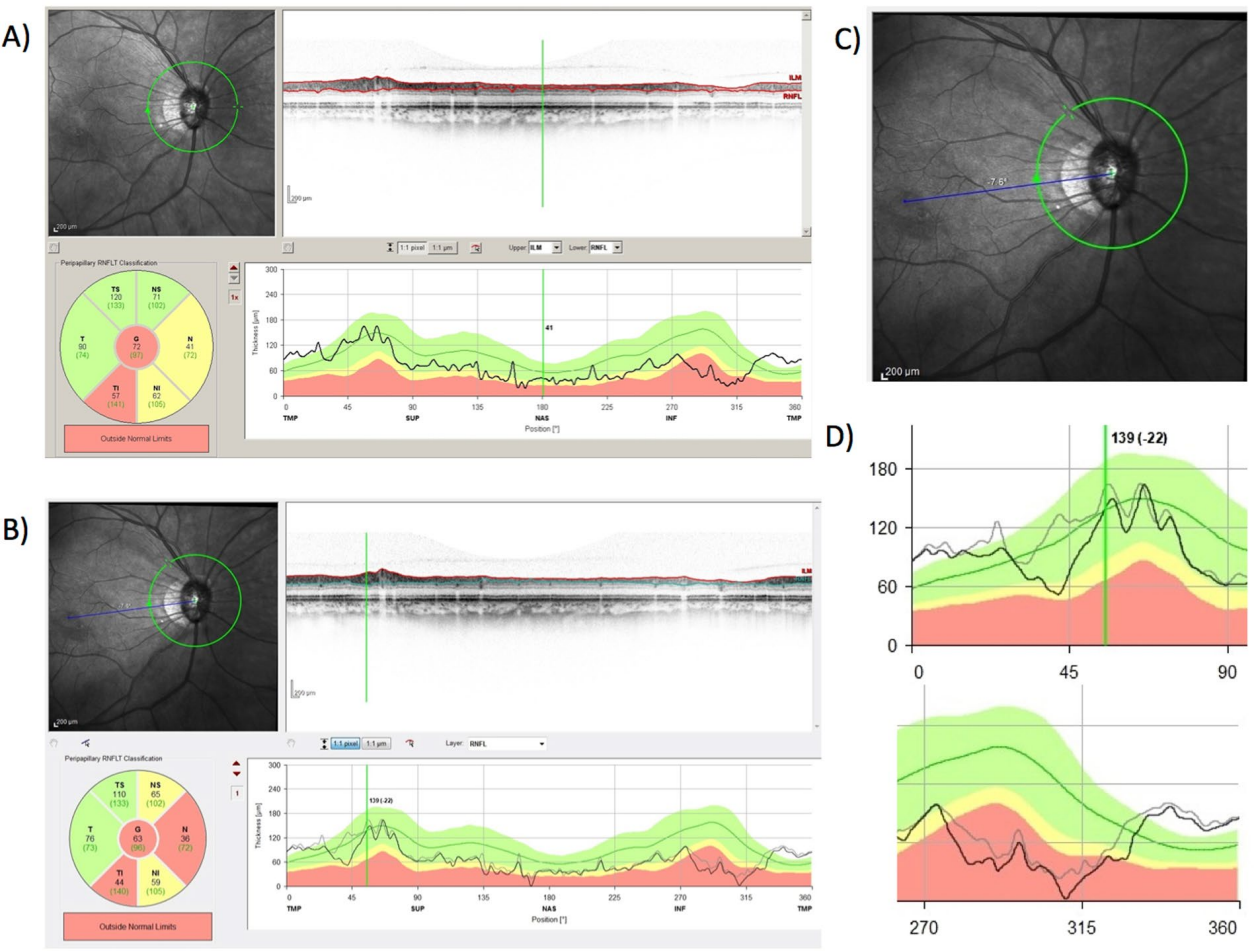
Measurement of the circumpapillary retinal nerve fiber layer thickness (cpRNFL thickness, or cpRNFLT) using the Spectralis follow-up mode. (**A**) Measurement at baseline. The infrared (IR) image of the disc at the upper left of the window, the original optical coherence tomography (OCT) image of the cpRNFL at the upper right, the global and sectorial thickness at the lower left, and the profile of the cpRNFL for comparison with the normative database at the lower right. (**B**) Measurement at follow-up. (**C**) Magnified IR image of the disc in (**B**). To examine the same location, the IR image was rotated slightly (see the upper right corner of the image). (**D**) The magnified image of the cpRNFL profile from the temporal to the superior sector. The gray line indicates the profile at the baseline; the black line at follow-up. The change of the profile at approximately 45 degrees supports the development of the NFLD observed in (**C**). (**E**) Magnified image of the cpRNFL profile from the inferior to the temporal sector. A comparison of the gray line (baseline cpRNFL) and the black line (follow-up cpRNFL) indicates the widening and deepening of the NFLD, which is not clearly visible in (**C**).

A reason for this disagreement may be the test-retest variability of OCT^14,15^. The measurement of retinal thickness using OCT also includes factors that may cause testing variability, including segmentation error^16^, signal strength^17^ and misplacement of the measurement location^18^. Recent advantages of spectral-domain OCT (SD-OCT) have enabled a reduction in the testing variability of these factors. In particular, the average scan of the Spectralis HRA + OCT system (Heidelberg Engineering, Heidelberg, Germany) reduces the influences of signal strength^19^ and segmentation error. In addition, the measurement of circumpapillary retinal nerve fiber layer thickness (cpRNFL thickness, or cpRNFLT) using an eye-tracking system (follow-up mode) enables measurement at approximately the same location as previous examinations (Fig. 1)^20,21^. Although the usefulness of the follow-up mode in glaucoma research has not been explicitly reported, Hood *et al*. articulately visualized the potential of the detection of glaucoma progression by evaluating changes in cpRNFL^22^. They showed that a comparison of cpRNFL scans from two visits could detect the widening of the nerve fiber layer defect after alignment. However, their report included a limited number of patients and did not compare the correlation with visual field (VF) progression.

**Figure 2:**
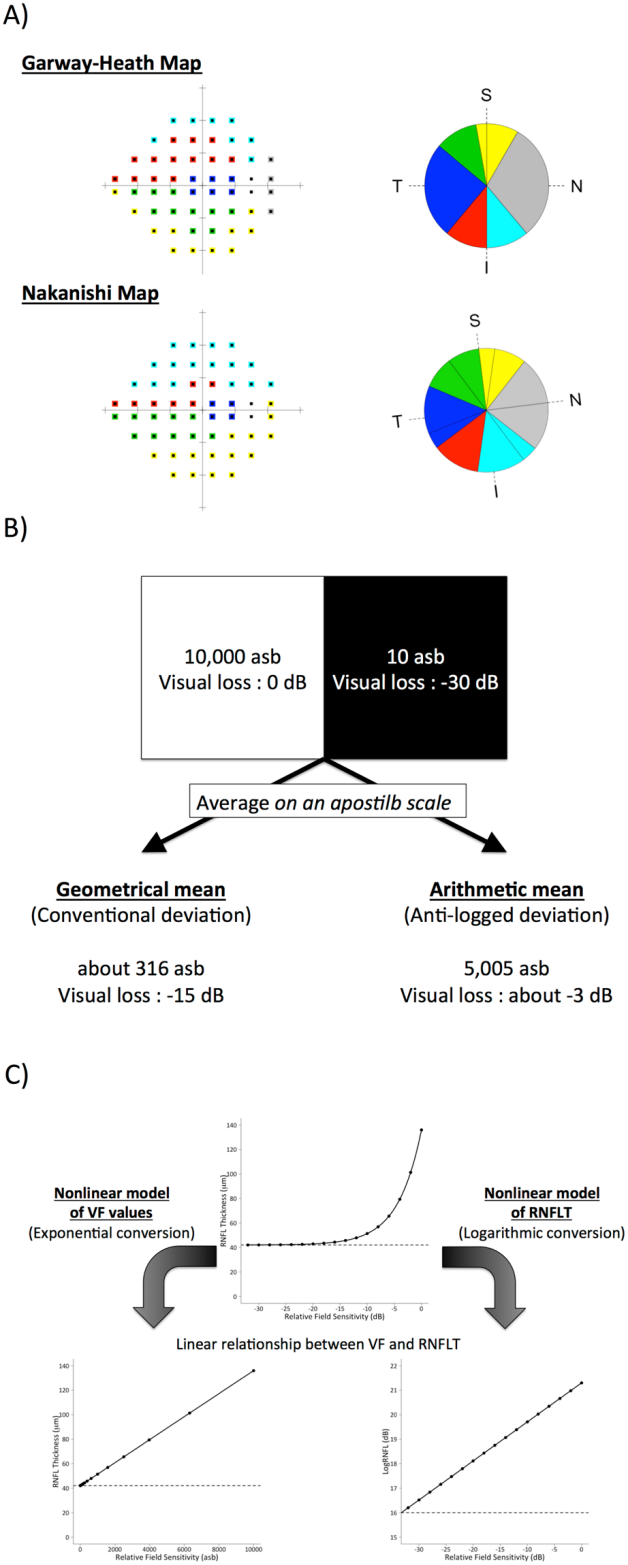
Complicated relationships between structure and function in glaucoma. (**A**) Structure-function relationship map; sectors with the same color correspond to each other. (**B**) Calculation of sectorial sensitivity of the visual field (VF). (**C**) Nonlinear relationship between the VF and the retinal nerve fiber layer thickness (RNFLT). If the VF values are converted to anti-log values (or if the RNFLT is converted to a logarithmic scale), the relationship becomes linear.

Another reason is the complex relationship between structure and function. A number of cross-sectional studies have revealed a detailed correlation between sensitivity in the sectional visual field and the thickness of the corresponding sectorial cpRNFLT^23^. The most famous idea is the Garway-Heath map (Fig. 2A)^24^, which associates OCT measurements and SAP results. We have reported another structure-function relationship map (Nakanishi map) based on clinical data collected at our glaucoma clinic^25^.

Another attempt to clarify the structure-function relationship in glaucoma is discussed in a review by Hood *et al*.^26^. Their argument consists of two issues. The first is how to calculate the sensitivity of sectorial visual field. They insist that the mean of the deviations should be calculated after each value is converted to the anti-log (Fig. 2B). The second concerns the nonlinear relationship between SAP and OCT values (Fig. 2C). In their cross-sectional clinical study, the exponential conversion of SAP values (or the logarithmic conversion of the sectorial cpRNFLT values) resulted in a linear relationship between SAP testing and OCT measurement^27^.

In this study, we investigated whether the application of the ideas and hypotheses (Fig. 2) discussed above could improve the consistency between longitudinal changes in SAP testing and OCT measurement. We also evaluated the ideas and hypotheses that improved the structure-function relationship in longitudinal changes in glaucoma.

## Results

We obtained informed consent from 230 glaucoma patients for enrollment in this longitudinal study. We excluded 51 patients (3 with diabetes mellitus; 1 with macular disease [central serous chorioretinopathy]; 4 with retinal tears; 2 with angle closure; 3 with steroid use; 17 with peripapillary atrophy too large to measure cpRNFLT; and 21 with epiretinal membrane or retinoschisis). We also excluded 48 patients because we could not get four reliable examinations of both SAP and Spectralis OCT. Finally, 131 eyes of 100 glaucomatous patients were included in the following analysis. The characteristics of the subjects are summarized in Table 1.

**Table 1:** Demographics.

Parameter	Mean ± Standard deviation	(Range)
Age (year)	53.2 ± 12.4	(21.7–77.4)
Right eye / Left eye	61/70	
Sex (Male / Female) (eye)	52/79	
Refractive error (diopter)	−4.96 ± 3.84	(−13.50–2.75)
Axial length (mm)	25.7 ± 1.57	(22.1–29.6)
SAP MD value at entry (dB)	−6.20 ± 4.34	(−19.5–0.21)
Number of SAP examinations	7.79 ± 3.57	(4–17)
Follow up period of SAP (year)	3.68 ± 0.947	(1.50–6.37)
Spectralis cpRNFL thickness at entry (µm)	68.5 ± 11.6	(36–96)
Number of Spectralis examinations	8.22 ± 3.03	(4–17)
Follow up period of Spectralis examinations (year)	3.69 ± 0.928	(1.50–6.62)
Fovea-ONH center angle, deg†	−7.63 ± 4.04	(−19.3–2.5)

^†^The angle between the fovea-ONH center axis and the horizontal axes.

The primary results of the linear mixed models are shown in Table 2. The estimated average baseline of SAP MD was −6.16 ± 0.39 dB (95% CI, −6.93 to −5.40 dB); the rate of SAP MD change was −0.30 ± 0.04 dB/year (95% CI, −0.38 to −0.22 dB/year). The estimated average baseline of the Spectralis global cpRNFLT was 68.72 ± 1.10 µm (95% CI, 66.56 to 70.89 µm); the rate of global cpRNFLT change was −0.95 ± 0.07 µm/year (95% CI, −1.08 to −0.81 µm/year). The linear mixed models detected significant negative rates of change in 6 sectors of the Spectralis cpRNFLT. We also analyzed the linear mixed models on the sectors of sensitivity thresholds and total deviations in SAP and of cpRNFLT in Spectralis OCT defined by the structure-function relationship maps. These results are shown in Supplemental Tables 1–3. In all sectors, the significant longitudinal changes were detected.

**Table 2:** Linear mixed models in this study.

Value		Mean ± SD	95% CI
SAP (dB)			
Mean deviation	Baseline β_0_	−6.16 ± 0.39	−6.93–−5.40
	Time β_1_ (year)	−0.30 ± 0.04	−0.38–−0.22
Pattern standard deviation	Baseline β_0_	7.94 ± 0.36	7.24–8.63
	Time β_1_ (year)	0.24 ± 0.04	0.16–0.33
Spectralis cpRNFLT (µm)			
Global	Baseline β_0_	68.72 ± 1.10	66.56–70.89
	Time β_1_ (year)	−0.95 ± 0.07	−1.08–−0.81
Temporal	Baseline β_0_	64.68 ± 1.72	61.31–68.04
	Time β_1_ (year)	−0.92 ± 0.12	−1.15–−0.69
Temporal superior	Baseline β_0_	88.81 ± 2.71	83.51–94.12
	Time β_1_ (year)	−1.43 ± 0.15	−1.73–−1.13
Temporal inferior	Baseline β_0_	73.35 ± 2.88	67.70–79.00
	Time β_1_ (year)	−1.67 ± 0.16	−1.98–−1.36
Nasal	Baseline β_0_	51.06 ± 1.60	47.92–54.21
	Time β_1_ (year)	−0.48 ± 0.09	−0.65–−0.30
Nasal superior	Baseline β_0_	80.45 ± 1.84	76.84–84.06
	Time β_1_ (year)	−0.89 ± 0.12	−1.12–−0.66
Nasal inferior	Baseline β_0_	75.32 ± 1.95	71.50–79.15
	Time β_1_ (year)	−0.86 ± 0.12	−1.09–−0.62

**Figure 3:**
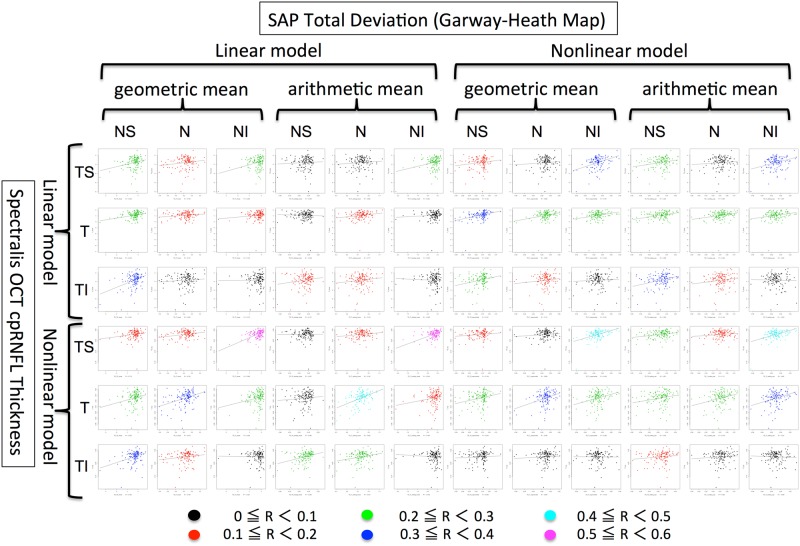
Correlation in sectorial longitudinal changes between standard automated perimetry (SAP) indices (total deviation) and circumpapillary retinal nerve fiber layer thickness (cpRNFLT) in the Garway-Heath map. The values of each sector were calculated considering the complex relationship between structure and function. The color of the points in the scatter plots indicates the strength of the correlation. Abbreviations: NI, nasal inferior; N, nasal; NS, nasal superior; TI, temporal inferior; T, temporal; TS, temporal superior.

To evaluate the correlation of longitudinal changes between VF and cpRNFL thickness, the baseline and rate of change of each sector of SAP or cpRNFLT in each eye were estimated by BLUPs, followed by visualization of the correlation by scatter plots and calculation of the Pearson’s correlation efficient using the BLUPs of the VF and cpRNFLT sectors. The scatter plots of the BLUPs of the SAP MD and the global cpRNFLT are shown in Supplemantal Fig. 1. The correlation coefficients of the estimated baseline and the rate of change were 0.548 (moderate relationship) and 0.358 (weak relationship), respectively. To increase the correlation between VF and cpRNFLT, we introduced three methods (Fig. 2): the structure-function relationship map (Garway-Heath map and Nakanishi map), the arithmetic mean of the sector VF, and a nonlinear model of VF and cpRNFLT. The results of scatter plot analysis after introducing these methods are shown in Fig. 3 and Supplemental Figs. 2–5. In terms of structure-function relationship map, analysis of the rates of change (Fig. 3 and Supplemental Fig. 3B) revealed that the correlation coefficients between corresponding sectors in the Garway-heath map were significantly higher than those between non-corresponding sectors (P < 0.01, Wilcoxon test).

**Figure 4:**
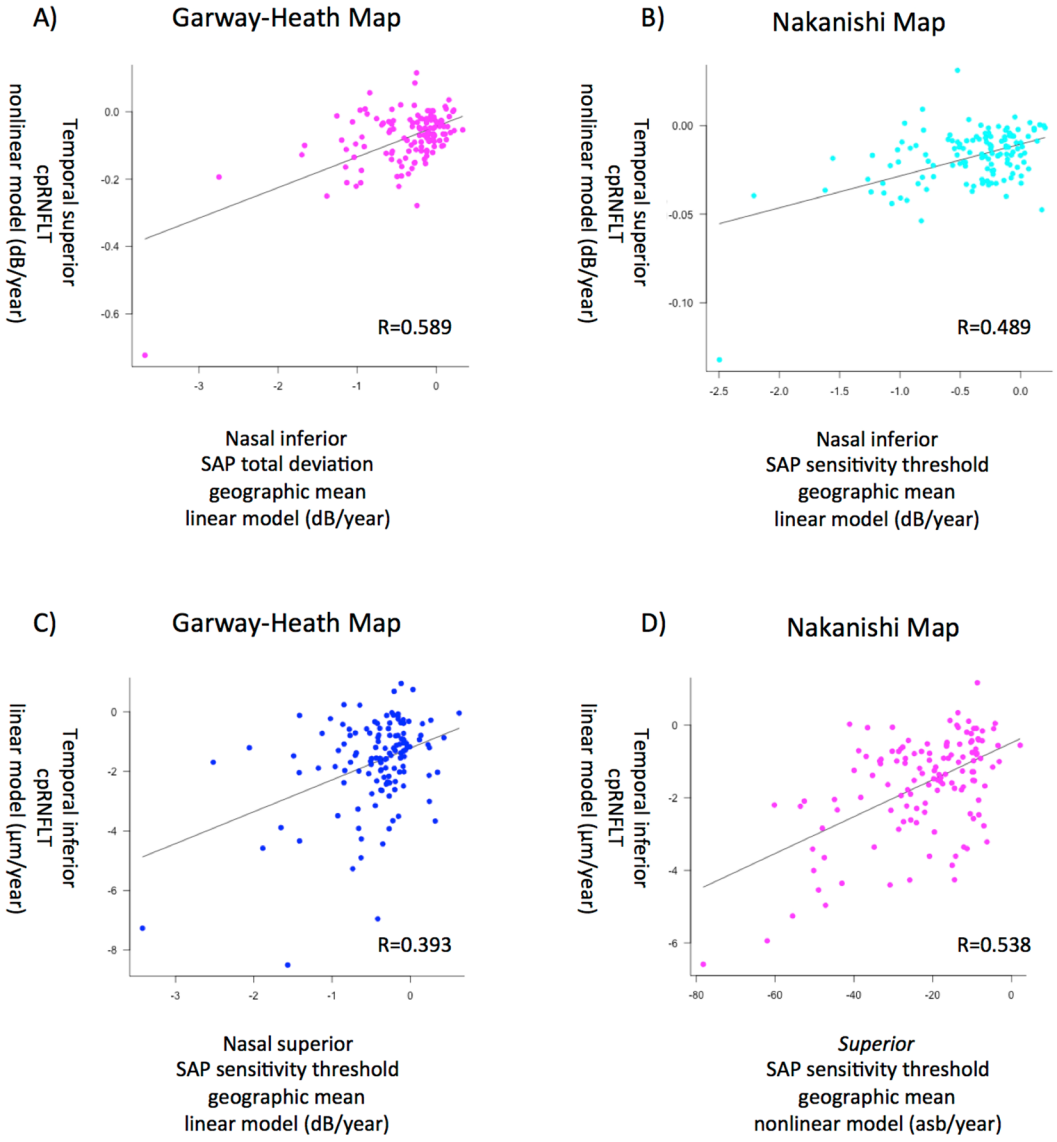
The scatter plots with the strongest correlation between the sectorial indices of standard automated perimetry (SAP) and circumpapillary retinal nerve fiber layer thickness (cpRNFLT) in each structure-function relationship map. (**A**) The scatter plot in the inferior sector of SAP (superior sector of cpRNFLT) in the Garway-Heath map. (**B**) The scatter plot in the inferior sector of SAP (superior sector of cpRNFLT) in the Nakanishi map. (**C**) The scatter plot in the superior sector of SAP (inferior sector of the cpRNFLT) in the Garway-Heath map. (**D**) The scatter plot in the superior sector of SAP (inferior sector of cpRNFLT) in the Nakanishi map.

The baselines of the scatter plots (Supplemental Figs 2 and 3A) revealed that the introduction of a nonlinear model to either SAP or cpRNFLT straightened the logarithmic correlation curve. Although in the analysis of rates of change (Fig. 3 and Supplemental Fig. 3B) it did not increase the correlation coefficients significantly (P = 0.077, Wilcoxon test), nonlinear models succeeded in improving the structure-functional correlation of longitudinal changes especially in the nasal inferior sector of the SAP TD with a nonlinear model of the Garway-Heath map (temporal superior sector of cpRNFLT in the linear model); the correlation coefficient of longitudinal evaluations in this sector of these models was 0.589. In the superior sector of the SAP (inferior sector of the cpRNFLT), the highest correlation coefficient was 0.538 between superior SAP sensitivity threshold (nonlinear model; Nakanishi map) and temporal inferior cpRNFLT (linear model). The scatter plots with the highest correlation coefficient in the superior or inferior sector in each structure-function relationship map are shown in Fig. 4. In terms of calculation of sectorial sensitivity of VF, arithmetic mean did not significantly improve the correlation compared with geometrical (conventional) mean in Fig. 3 and Supplemental Fig. 3B (P = 0.37, Wilcoxon test).

## Discussion

In this study, we investigated the correlation of the longitudinal changes between the SAP indices and the cpRNFLT measured using Spectralis Follow-up mode. Comparing our previous report^19^, the correlation coefficiency of the rate of change between SAP MD and cpRNFLT became higher by introducing Follow-up mode (R = 0.358, with Follow-up mode; R = 0.16 without Follow-up mode). Moreover, the introduction of the structure-function relationship map and nonlinear relationship between the VF and cpRNFLT improved the correlation between the SAP indices and cpRNFLT in corresponding sectors (R = 0.589 or 0.538; compare Fig. 4 with Supplemental Fig. 1). These results justify not only the application of those two ideas (structure-function relationship map and nonlinear relationship in Fig. 2) in considering the structure-function relationship in longitudinal studies but also practical use of OCT in evaluating the glaucoma progression: progressive thinning of cpRNFL indicate deterioration of visual field defects.

The usefulness of introducing structure-function relationship map was expected for the following reasons; the focal structural or functional change in glaucoma is not necessarily followed by changes in other regions, as glaucoma is characterized by focal damage of the visual field and retinal nerve fiber layer (nasal step, Bjerrum scotoma, focal rim thinning or nerve fiber layer defect [NFLD]), not by their global deterioration^1,28,29^. Previous studies of the structure-function relationship in glaucoma progression^11,12^ that compared the global indices revealed a relationship; the mean deviation in Humphrey 24-2 only corresponded with the temporal side of the cpRNFL. Thus, recent studies have also focused on progressive RNFL thinning as determined by event analysis (Guided Progression Analysis [GPA]), using the RNFL thickness map acquired by SD-OCT^30,31^. Although this strategy is informative for the discrete evaluation of progression, the detailed relationship between longitudinal changes of VF and cpRNFL remains to be determined (We will discuss the problem of event analysis later).

We used two types of structure-function relationship map in this study: the Garway-Heath map and the Nakanishi map. While the nasal inferior sector of the Garway-Heath map was correlated most strongly with the temporal superior sector of the cpRNFLT, the strongest correlation was in the superior sector of visual field came from combination with the superior sector of VF and the temporal inferior (not inferior) sector of cpRNFLT in Nakanishi map. This result replicated the correlation in the original cross-sectional report from Nakanishi *et al*.^25^. Hood *et al*. depicted the schematic model of the glaucomatous damage of the macula, indicating that retinal ganglion cells within “vulnerable region” in the inferior visual field including macula project to the inferior quadrant of the disc (corresponding to the temporal inferior sector of cpRNFLT in Nakanishi map)^32^. Our results may support Hood’s schematic model even in the longitudinal study. While the correlation differed according to the structure-function relationship map, we could not determine the superiority of either map because this study included cases that were also included when creating the Nakanishi map. As described in Table 1, the participants in this study were myopic like those in the studies from Asia and may have different characteristics from those who contributed to induce Garway-Heath map.

The application of the nonlinear relationship between the VF and cpRNFLT also improved the correlation of the evaluations of the longitudinal changes in some sectors, indicating that the disagreement between the VF and RNFLT in previous longitudinal studies may have been due to the hypothesis that the longitudinal relationship between the VF and the RNFLT is linear. The nonlinear relationship implies that the simple application of event analysis to both VF and RNFLT may mislead judgment. In the detection of glaucoma progression, the baseline of the VF or RNFLT should be considered. The thinning of thick RNFL rarely affects the visual field, but slight changes in thin RNFL may lead to remarkable VF deterioration. The famous continuum proposed by Weinreb insisted that structural changes precede functional changes^33^. Abe *et al*. also showed in a longitudinal study that the detection rate of progression by SD-OCT (but not by SAP) was higher in eyes with less severe disease. An increase in disease severity increased the chance of detection by SAP, but not SD-OCT^34^. The nonlinear model between SAP and cpRNFLT supported their hypothesis and reports. We should consider the disease severity regardless of the use of SAP or SD-OCT to detect glaucoma progression.

We also evaluated a way to calculate the average sensitivity or deviation of the SAP sector, but the difference was not significant. According to Weber-Fechner’s law^35^, SAP indices should be calculated on a logarithmic scale, as visual acuity is. This means Weber-Fechner’s law also supports the conventional method of the use of the mean, total deviation, or MD slope. Our results could not determine the correctness of Weber-Fechner’s law or resolve the debate between Harwerth and Hood^26,36^. It appears that it is more important to select the appropriate combination of methods to calculate the longitudinal changes both in SAP indices and cpRNFLT. Similarly, our results did not necessarily support the nonlinear regression model proposed by Pathak *et al*.^37^, because in this study changes in treatment were not prohibited. The introduction of a nonlinear model in this study was merely to improve the structure-function relationship in terms of longitudinal changes.

This study had several limitations. The first was the small sample size of both cases (patients) and experiments. To avoid the incorrect evaluation of longitudinal changes, we applied linear mixed effect models and BLUPs^38^, but a larger cohort may reveal the precise and detailed relationship between VF and RNFLT. The second was the exclusion of cases with ERM or retinoschisis from the study. As the strategy of this study relied on the hypothesis that decrease of RNFLT directly results in VF progression, we had to exclude other factors that could influence RNFLT without changing the VF. As stated in the first section of the Results, many glaucoma cases are accompanied by ERM or retinoschisis. Determination of the optimal evaluation method of the structural longitudinal changes in those cases is goal for future research. The third was the modeling used in this study. As previous studies have suggested^26^, cpRNFLT is never less than 30 µm even in advanced cases, as components other than the nerve fiber layer (vessels and glial tissues) remain^39^. Correlation of this floor effect to the model was not possible, as in some cases the sectorial thickness of the cpRNFL measured by the Spectralis OCT decreased to <10 µm. Another cross-sectional study could not detect the floor effect^40^, so we must verify its validity in future studies.

In conclusion, we evaluated the correlation between structural and functional changes of glaucoma in a longitudinal study. The results of this study support the practical use of OCT in glaucoma progression but also stress the importance of focus on the changes in the corresponding sectors and the consideration of disease severity. Future studies will reveal that other modalities to measure the structural changes are also correlated with functional changes or with those detected by cpRNFLT.

## Methods

This prospective longitudinal study adhered to the tenets of the Declaration of Helsinki and was approved by the Institutional Review Board and Ethics Committee of the Kyoto University Graduate School of Medicine. Study subjects were prospectively enrolled at Kyoto University Hospital between April 2008 and October 2016, and informed consent was obtained from all patients.

All patients included in this study had a normal angle on gonioscopy and a Snellen equivalent best-corrected visual acuity (BCVA) of 20/20 or better to ensure high imaging quality. Subjects also had a typical glaucomatous VF defect (as seen on SAP using the Swedish interactive threshold algorithm [SITA] 24-2) with corresponding optic disc changes (narrowing of the neuroretinal rim) and/or thinning of the retinal nerve fiber layer (RNFL), based on the Ocular Hypertension Treatment Study (OHTS) criteria^41^. Eyes were excluded from analysis if they had a cataract that could affect visual function, vitreoretinal disease, pathologic myopia with patchy chorioretinal atrophy, a lacquer crack lesion or choroidal neovascularization, uveitis, or prior ocular surgery including laser therapy. The use and changes of topical IOP-lowering agents was permitted during this study. Patients were also excluded from participation if they had a neurological disease, diabetes mellitus, or any other systemic disease that might affect the eye or the VF (such as a cerebrovascular event, uncontrolled hypertension, or blood disorders). Data from patients who developed an epiretinal membrane or retinoschisis on the macula or around the optic disc during follow-up were excluded because they could influence retinal thickness measurements. To avoid the introduction of selection bias, data from patients who underwent ocular surgery during the follow-up period were included until the date of surgery.

### Clinical examinations

All subjects underwent a comprehensive baseline ophthalmic examination, including measurement of IOP with a Goldman applanation tonometer and uncorrected and best-corrected visual acuity with a Landolt chart at 5 m. Subjects also underwent slit-lamp examination, gonioscopy, stereoscopic optic disc photography (3-Dx simultaneous stereo disc camera; Nidek, Gamagori, Japan), SAP with a Humphrey Visual Field Analyzer (Carl Zeiss-Meditec, Dublin, CA, USA) using the 24-2 SITA standard testing protocol (HFA + 24-2 SITA), and OCT examinations (Spectralis HRA + OCT system; Heidelberg Engineering, Heidelberg, Germany). All subjects had at least four VF and OCT examinations during the follow-up period. The first and last VF and OCT examinations were performed within 3 months.

#### Visual field examinations

Only reliable VF tests were used for analysis. The reliabilities were defined as the fixation loss (<20%), false-positive error rate, and false-negative rate (<33%). A glaucomatous VF defect was defined as (1) glaucoma hemifield test results outside normal limits; (2) more than three significant (P < 0.05) and one highly significant (P < 0.01) non-edge contiguous points on the same side of the horizontal meridian as in the pattern deviation plot; or (3) a pattern standard deviation (PSD) < 5% in an otherwise normal VF. All VF findings were confirmed in at least two consecutive testing sessions. To compensate for the subject’s learning process, the first VF test was not included in the analysis if it was the first VF test the subject had taken.

#### Spectral-domain optical coherence tomography imaging

The Spectralis HRA + OCT system was used to evaluate the cpRNFLT. The eye tracking system of this instrument allows accurate averaging of up to 100 B-scans (7 μm axial resolution) acquired at an identical location, which can efficiently reduce speckle noise, and enables measurement at approximately the same location during every examination. For cpRNFL imaging, we performed a 3.46 mm diameter circular scan centered on the optic disc and consisting of 1536 A-scans. A total of 16 scans were acquired and averaged to obtain the final scan used in analysis. For repeated examinations of cpRNFLT, only the measurements taken in follow-up mode were used, as they measured the same location as the first examination.

### Structure-function relationship map

To more accurately evaluate the sectorial relationship between SAP and cpRNFL thickness in OCT, we introduced the “structure-function relationship map.” In this study, we used the Garway-Heath map^24^ (most famous and widespread in glaucoma society) and the Nakanishi map (produced from a clinical study at our hospital; Fig. 2A)^25^. To calculate the sectorial cpRNFL thickness, we exported 768 values of the cpRNFLT along the 360-degree OCT scan using RNFL export software (Heidelberg Engineering). The arithmetic means of the values corresponding to each sector were used as the sectorial thickness of cpRNFL. For the Nakanishi map, in which the fovea-optic nerve head (ONH) center angle should be considered, the averaged values were chosen after adjustment for the influence of the fovea-ONH angle measured using the built-in software. In SAP, the sectorial threshold sensitivities or total deviations (TDs) were also calculated from the means of the values corresponding to the defined sectors in the map. In this study, the means of the VF values were calculated on an apostilb scale both arithmetically (anti-log TD) and geometrically (arithmetically on a dB scale; conventional TD), as it remains controversial whether the total deviation values should be anti-logged before averaging^26^. Both types of means were analyzed.

### Nonlinear relationship between structure and function

When comparing the longitudinal changes of the VF and OCT results, the nonlinear relationship between structure and function in the cross section must be considered. To correct the nonlinear relationship to a linear one, the sectorial means of the VF values (threshold sensitivities and total deviations) were converted to exponential values (1/Lambert [1/L] scale) using the following formula:

1 $${\rm{dB}}={\rm{10}}\times {\mathrm{log}}_{{\rm{10}}}(1/{\rm{L}})={\mathrm{log}}_{{\rm{1.259}}}(1/{\rm{L}})$$

Similarly, the sectorial means of cpRNFL thickness were converted to logarithmic values (defined as LogRNFLT in this study):

2 $${\rm{LogRNFLT}}={\rm{10}}\times {\mathrm{log}}_{{\rm{10}}}({\rm{cpRNFLT}})={\mathrm{log}}_{{\rm{1.259}}}({\rm{cpRNFLT}})$$

These exponential VF values (1/L) and logarithmic OCT values (LogRNFL) were also used to analyze the longitudinal change in each case as nonlinear models. The unconverted values were used in linear models in the next analysis (described in the next subsection).

### Statistical analysis

The longitudinal time trends of the outcome measures were evaluated using linear mixed models fitted with random intercepts and coefficients at both the subject and eye levels. Linear mixed-effects modeling allowed the determination of correlations among repeated measurements and the use of two eyes from the same subject^38^. Linear mixed-effects models can also manage data sets with multiple missing data points or with high variation in examination times. The following equation describes the corrections applied to our data.

3 $${Y}_{ijt}={\beta }_{0}+{\beta }_{1}\,{\rm{TIME}}+{\zeta }_{0j}+{\zeta }_{1j}\times {\rm{TIME}}+{\zeta }_{0i|j}+{\zeta }_{1i|j}\times {\rm{TIME}}+{{\rm{\varepsilon }}}_{ijt}$$

where *Y*_*ijt*_ is the individual measurement at visit *t*; *β*_0_ *and β*_1_ are the fixed-effects coefficients; *ζ*_0*j*_ and *ζ*_1*j*_ are the random patient effects associated with the intercept and time slope; and *ζ*_0*i*|*j*_ and *ζ*_1*i*|*j*_ are the random effects associated with including both eyes of a single subject.

As we evaluated the simple relationship of the longitudinal changes between the VF values and cpRNFL thickness, as we usually do in clinical situations, we did not enter other fixed effects such as age, axial length, or imaging quality.

To evaluate the individual heterogeneity of baselines and time trends in each examined eye, estimates of best linear unbiased predictors (BLUPs) were calculated and analyzed in this linear mixed model. The relationships between VF and OCT cpRNFL in longitudinal changes were evaluated by correlation coefficients calculated from the BLUPs of the VF values (arithmetic or geometrical means × linear or exponential values = 4 patterns) and OCT values (linear or logarithmic values). To visualize the strength of the correlation coefficients, the color of the dots in the scatter plot varied according to the strength of the correlation coefficient (Fig. 3; Supplemental Figs 2–5).

All P-values presented are two-sided values. The statistical significance was defined as P < 0.05. The differences of correlation coefficients in each model (structure-function relationship map, nonlinear model and calculation of mean deviation. Also see Fig. 2) were compared using the Wilcoxon signed rank test. All analyses were performed using R ver. 3.3.2 (R Foundation for Statistical Computing, Vienna, Austria) and SAS ver. 9.4 (SAS Institute, Inc, Cary, NC) statistical software.

## Electronic supplementary material

Dataset1

Dataset2

Dataset3

Dataset4

Supplemental Information

## Data Availability

We uploaded the raw data used in this work as Supplement Dataset with the manuscript.
