# Supplementary material for: Evaluation of Structure-Function Relationships in Longitudinal Changes of Glaucoma using the Spectralis OCT Follow-Up Mode
Source: Sci Rep. 2018 Nov 21;8:17158. doi: 10.1038/s41598-018-35419-y (PMC6249277; doi:10.1038/s41598-018-35419-y)
Supplement: Supplementary file 5 — Supplemental Information [file 41598_2018_35419_MOESM5_ESM.pdf]

Supplementary Information

Evaluation of Structure-Function Relationships in Longitudinal Changes of Glaucoma using the Spectralis OCT Follow-Up Mode

Kenji Suda[1)], Tadamichi Akagi[1)], Hideo Nakanishi[1)], Hisashi Noma[2)], Hanako Ohashi Ikeda[1)], Takanori Kameda[1)], Tomoko Hasegawa[1)], and Akitaka Tsujikawa[1)]

1) Department of Ophthalmology and Visual Sciences, Kyoto University Graduate School of Medicine, 54 Kawahara-cho, Shogoin, Sakyo-ku, Kyoto 606-8507, Japan
2) Department of Data Science, The Institute of Statistical Mathematics, 10-3 Midori-cho, Tachikawa, Tokyo 190-8562, Japan

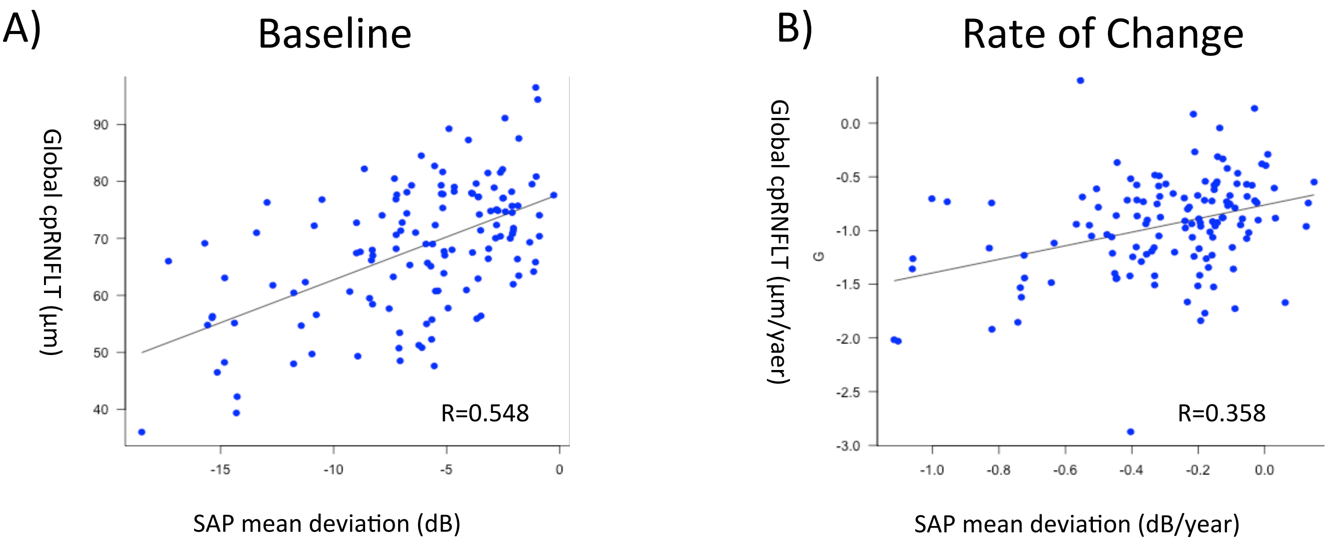

Supplemental Figure 1. Correlations in the baseline and longitudinal changes between the global indices of standard automated perimetry (SAP) and circumpapillary retinal nerve fiber layer thickness (cpRNFLT). Each point in the scatter plots indicates the best linear unbiased predictors (BLUPs) of each case calculated by linear mixed models. The correlation of the baseline is moderately strong (R = 0.548), but the correlation of the longitudinal change is weak (R = 0.358).

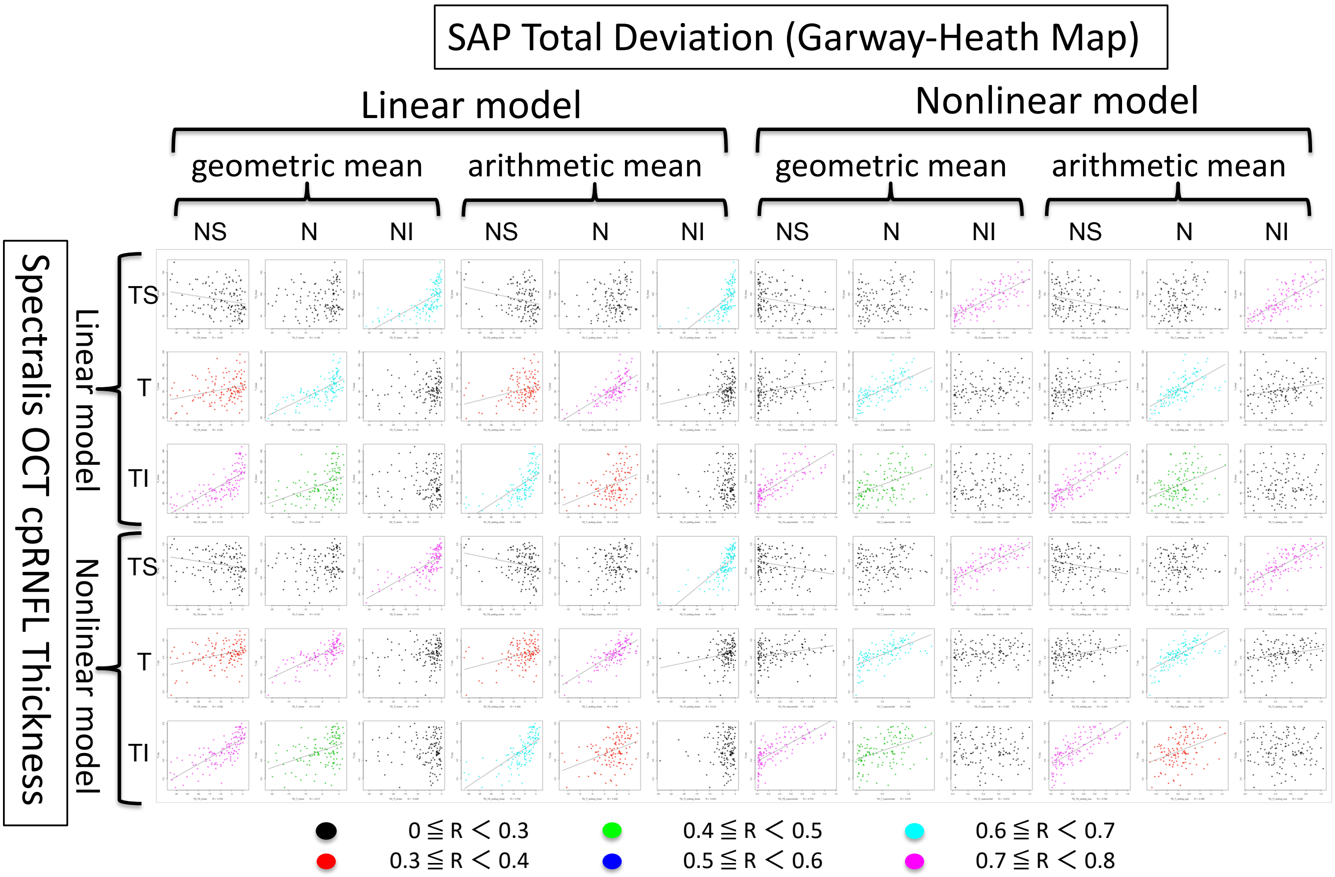

Supplemental Figure 2. Correlation in the sectorial baselines between the indices of standard automated perimetry (SAP) (total deviation) and circumpapillary retinal nerve fiber layer thickness (cpRNFLT) in the Garway-Heath map. The values of each sector were calculated in consideration of the complex relationship between structure and function. The color of the points in the scatter plots indicates the strength of the correlation. Abbreviations: NI, nasal inferior; N, nasal; NS, nasal superior; TI, temporal inferior; T, temporal; TS, temporal superior.

A)

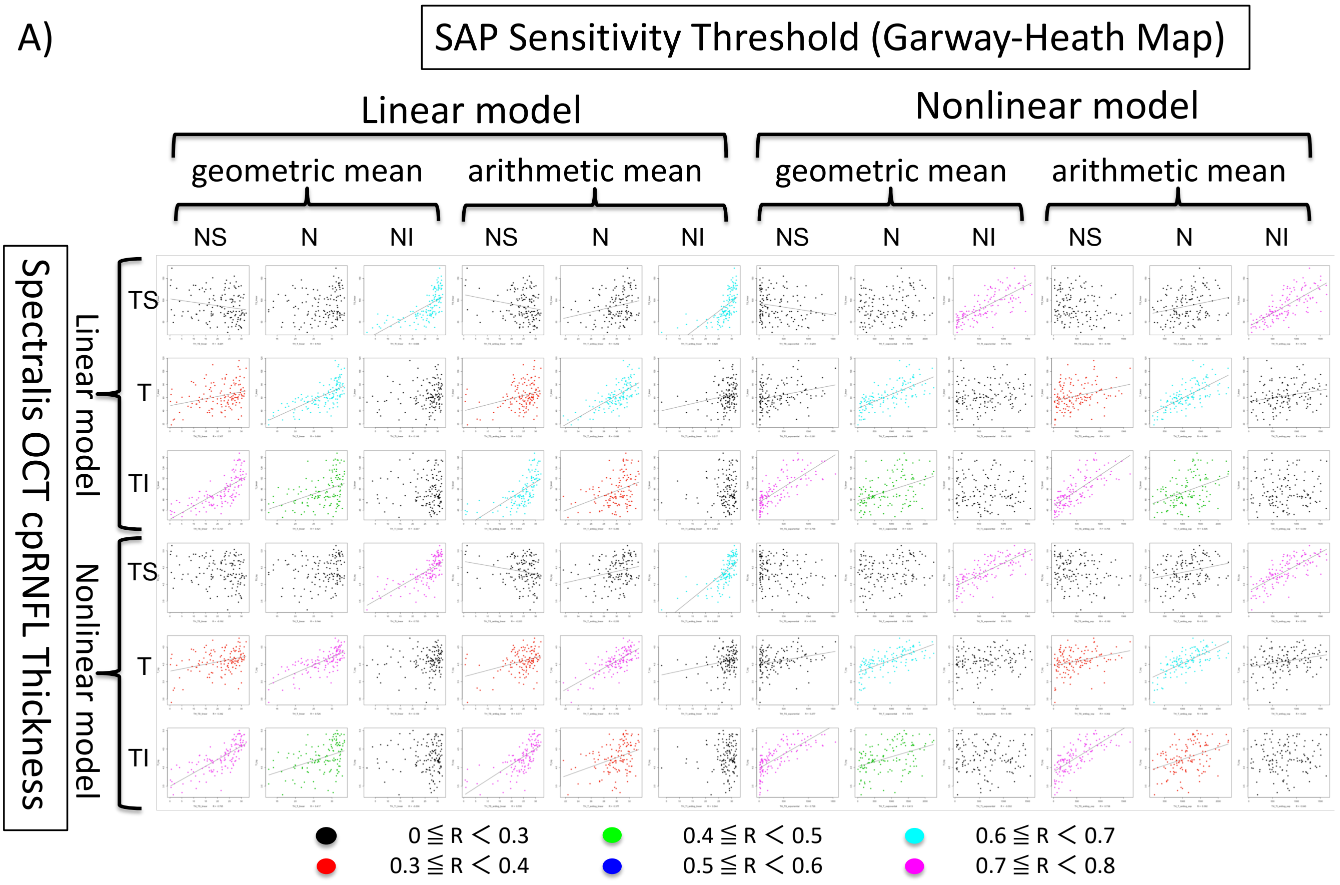
SAP Sensitivity Threshold (Garway-Heath Map)
Linear model
Nonlinear model
geometric mean
arithmetic mean
geometric mean
arithmetic mean
NS
N
NI
NS
N
NI
NS
N
NI
NS
N
NI
Spectralis OCT cpRNFL Thickness
Linear model
Nonlinear model
TS
T
TI
TS
T
TI
0 ≦ R < 0.3
0.3 ≦ R < 0.4
0.4 ≦ R < 0.5
0.5 ≦ R < 0.6
0.6 ≦ R < 0.7
0.7 ≦ R < 0.8

B)

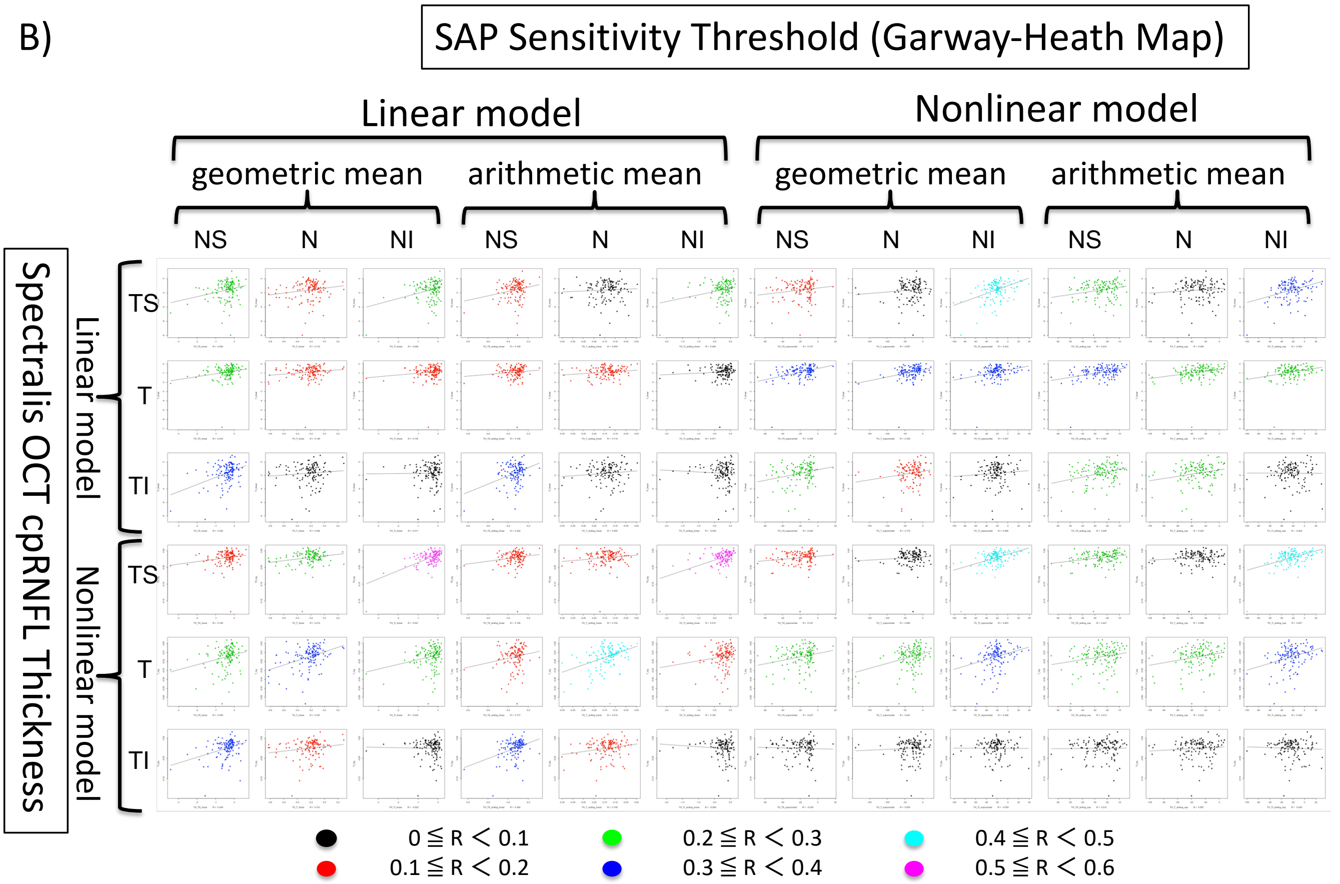
SAP Sensitivity Threshold (Garway-Heath Map)
Linear model
Nonlinear model
geometric mean
arithmetic mean
geometric mean
arithmetic mean
NS
N
NI
NS
N
NI
NS
N
NI
NS
N
NI
Spectralis OCT cpRNFL Thickness
Linear model
Nonlinear model
TS
T
TI
TS
T
TI
0 ≦ R < 0.1
0.1 ≦ R < 0.2
0.2 ≦ R < 0.3
0.3 ≦ R < 0.4
0.4 ≦ R < 0.5
0.5 ≦ R < 0.6

Supplemental Figure 3. Correlations in the sectorial baselines and longitudinal changes between the indices of standard automated perimetry (SAP) (sensitivity threshold) and circumpapillary retinal nerve fiber layer thickness (cpRNFLT) in the Garway-Heath map. The values of each sector were calculated considering the complex relationship between structure and function. The color of the points in the scatter plots indicates the strength of the correlation. (A) Correlation of sectorial baselines. (B) Correlation of sectorial longitudinal changes.

A)

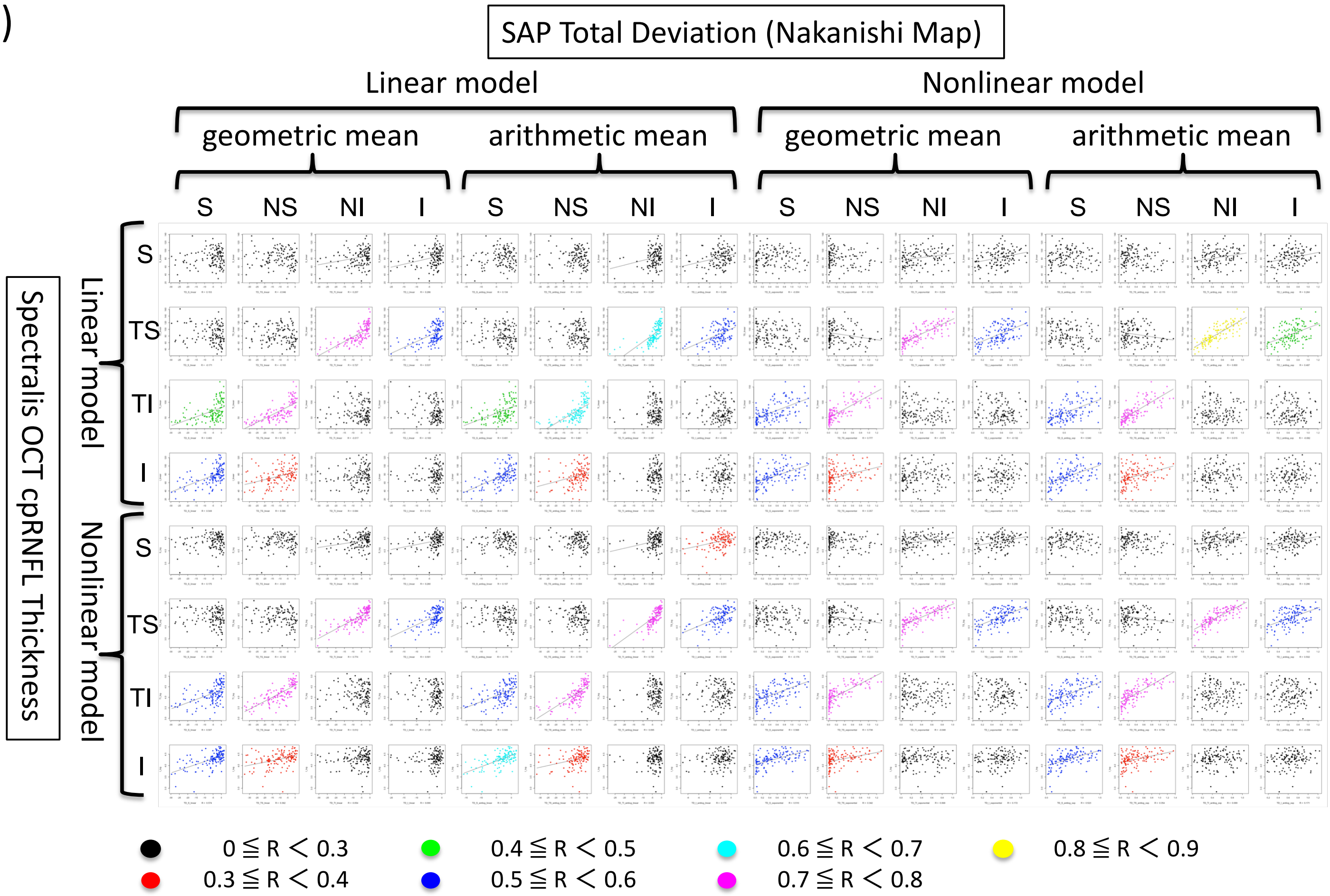
SAP Total Deviation (Nakanishi Map)
Linear model
Nonlinear model
geometric mean
arithmetic mean
geometric mean
arithmetic mean
S NS NI I S NS NI I S NS NI I S NS NI I
Spectralis OCT cpRNFL Thickness
Linear model
S
TS
TI
I
Nonlinear model
S
TS
TI
I
0 ≦ R ＜ 0.3
0.3 ≦ R ＜ 0.4
0.4 ≦ R ＜ 0.5
0.5 ≦ R ＜ 0.6
0.6 ≦ R ＜ 0.7
0.7 ≦ R ＜ 0.8
0.8 ≦ R ＜ 0.9

B)

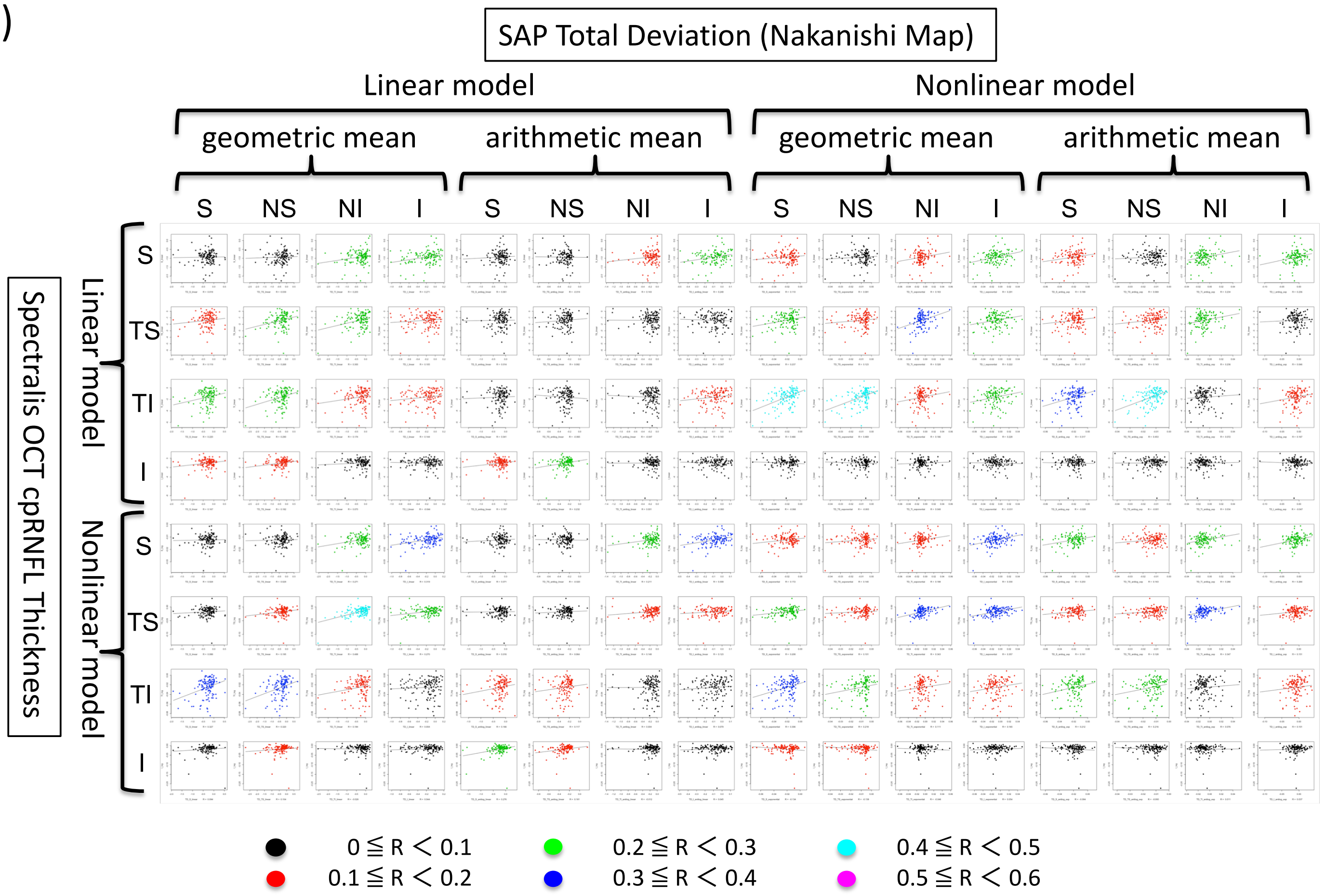
SAP Total Deviation (Nakanishi Map)
Linear model
Nonlinear model
geometric mean
arithmetic mean
geometric mean
arithmetic mean
S NS NI I S NS NI I S NS NI I S NS NI I
Spectralis OCT cpRNFL Thickness
Linear model
S
TS
TI
I
Nonlinear model
S
TS
TI
I
0 ≦ R ＜ 0.1
0.1 ≦ R ＜ 0.2
0.2 ≦ R ＜ 0.3
0.3 ≦ R ＜ 0.4
0.4 ≦ R ＜ 0.5
0.5 ≦ R ＜ 0.6

Supplemental Figure 4. Correlations of the sectorial baselines and longitudinal changes between indices of standard automated perimetry (SAP) (total deviation) and circumpapillary retinal nerve fiber layer thickness (cpRNFLT) in the Nakanishi map. The values of each sector were calculated considering the complex relationship between structure and function. The color of the points in the scatter plots indicates the strength of the correlation. (A) Correlation of the sectorial baselines. (B) Correlation of the sectorial longitudinal changes.

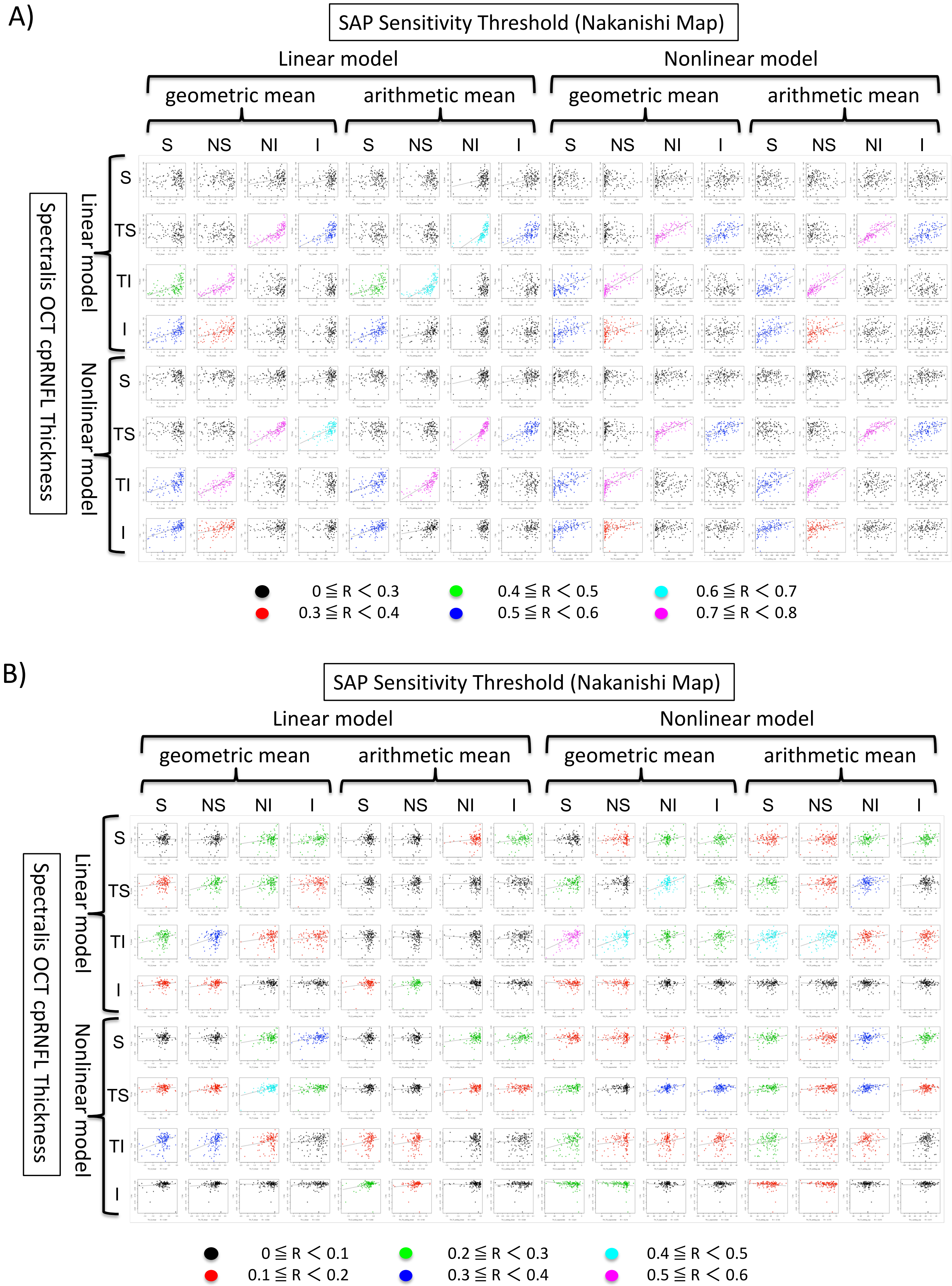
A)
SAP Sensitivity Threshold (Nakanishi Map)
Linear model
Nonlinear model
geometric mean
arithmetic mean
geometric mean
arithmetic mean
S NS NI I S NS NI I S NS NI I S NS NI I
Spectralis OCT cpRNFL Thickness
Linear model
S
TS
TI
I
Nonlinear model
S
TS
TI
I
0 ≦ R < 0.3
0.3 ≦ R < 0.4
0.4 ≦ R < 0.5
0.5 ≦ R < 0.6
0.6 ≦ R < 0.7
0.7 ≦ R < 0.8
B)
SAP Sensitivity Threshold (Nakanishi Map)
Linear model
Nonlinear model
geometric mean
arithmetic mean
geometric mean
arithmetic mean
S NS NI I S NS NI I S NS NI I S NS NI I
Spectralis OCT cpRNFL Thickness
Linear model
S
TS
TI
I
Nonlinear model
S
TS
TI
I
0 ≦ R < 0.1
0.1 ≦ R < 0.2
0.2 ≦ R < 0.3
0.3 ≦ R < 0.4
0.4 ≦ R < 0.5
0.5 ≦ R < 0.6

Supplemental Figure 5. Correlations of the sectorial baselines and longitudinal changes between the indices of standard automated perimetry (SAP) (sensitivity threshold) and circumpapillary retinal nerve fiber layer thickness (cpRNFLT) in the Nakanishi map. The values of each sector were calculated considering the complex relationship between structure and function. The color of the points in the scatter plots indicates the strength of the correlation. (A) Correlation of the sectorial baselines. (B) Correlation of the sectorial longitudinal changes.

Supplementary Table 1 Linear mixed models in Garway-Heath map

Value		Mean ± SD	95% CI
Geometrical mean (conventional threshold sensitibities), linear model			
Temporal superior	Baseline β_0	21.465 ± 0.777	19.941 - 22.989
	Time β_1 (year)	-0.432 ± 0.073	-0.575 - -0.289
Temporal	Baseline β_0	27.353 ± 0.492	26.388 - 28.318
	Time β_1 (year)	-0.234 ± 0.052	-0.336 - -0.132
Temporal inferior	Baseline β_0	25.429 ± 0.628	24.198 - 26.659
	Time β_1 (year)	-0.421 ± 0.075	-0.567 - -0.274
Arithmetic mean (anti-logged threshold sensitibities), linear model			
Temporal superior	Baseline β_0	24.437 ± 0.635	23.193 - 25.681
	Time β_1 (year)	-0.324 ± 0.048	-0.419 - -0.229
Temporal	Baseline β_0	29.911 ± 0.231	29.458 - 30.365
	Time β_1 (year)	-0.134 ± 0.027	-0.186 - -0.081
Temporal inferior	Baseline β_0	27.613 ± 0.418	26.793 - 28.432
	Time β_1 (year)	-0.289 ± 0.060	-0.407 - -0.171
Geometrical mean (conventional threshold sensitibities), nonlinear model			
Temporal superior	Baseline β_0	390.994 ± 36.642	319.177 - 462.811
	Time β_1 (year)	-19.750 ± 2.932	-25.496 - -14.005
Temporal	Baseline β_0	889.435 ± 58.727	774.33 - 1004.54
	Time β_1 (year)	-33.761 ± 6.811	-47.109 - -20.412
Temporal inferior	Baseline β_0	644.327 ± 42.817	560.408 - 728.247
	Time β_1 (year)	-21.436 ± 4.324	-29.911 - -12.961
Arithmetic mean (anti-logged threshold sensitibities), nonlinear model			
Temporal superior	Baseline β_0	501.737 ± 36.479	430.239 - 573.234
	Time β_1 (year)	-25.079 ± 2.843	-30.650 - -19.507
Temporal	Baseline β_0	1131.81 ± 47.59	1038.52 - 1225.09
	Time β_1 (year)	-31.132 ± 6.369	-43.614 - -18.650
Temporal inferior	Baseline β_0	767.598 ± 39.092	690.978 - 844.217
	Time β_1 (year)	-21.550 ± 4.483	-30.337 - -12.763
Geometrical mean (conventional total deviation), linear model			
Temporal superior	Baseline β_0	-9.069 ± 0.816	-10.668 - -7.470
	Time β_1 (year)	-0.383 ± 0.076	-0.532 - -0.234
Temporal	Baseline β_0	-5.170 ± 0.503	-6.155 - -4.184
	Time β_1 (year)	-0.175 ± 0.054	-0.280 - -0.069
Temporal inferior	Baseline β_0	-6.306 ± 0.634	-7.548 - -5.064

	Time β_1 (year)	-0.384 ± 0.077	-0.534	-	-0.233
Arithmetic mean (anti-logged total deviation), linear model					
Temporal superior	Baseline β_0	-6.128 ± 0.653	-7.408	-	-4.847
	Time β_1 (year)	-0.259 ± 0.046	-0.350	-	-0.169
Temporal	Baseline β_0	-2.564 ± 0.227	-3.010	-	-2.118
	Time β_1 (year)	-0.064 ± 0.027	-0.118	-	-0.011
Temporal inferior	Baseline β_0	-4.159 ± 0.410	-4.963	-	-3.356
	Time β_1 (year)	-0.232 ± 0.060	-0.349	-	-0.115
Geometrical mean (conventional total deviation), nonlinear model					
Temporal superior	Baseline β_0	0.363 ± 0.034	0.297	-	0.429
	Time β_1 (year)	-0.014 ± 0.003	-0.019	-	-0.008
Temporal	Baseline β_0	0.505 ± 0.033	0.441	-	0.569
	Time β_1 (year)	-0.011 ± 0.004	-0.019	-	-0.004
Temporal inferior	Baseline β_0	0.432 ± 0.027	0.378	-	0.485
	Time β_1 (year)	-0.010 ± 0.003	-0.017	-	-0.004
Arithmetic mean (anti-logged total deviation), nonlinear model					
Temporal superior	Baseline β_0	0.449 ± 0.033	0.385	-	0.514
	Time β_1 (year)	-0.017 ± 0.003	-0.022	-	-0.012
Temporal	Baseline β_0	0.636 ± 0.026	0.584	-	0.688
	Time β_1 (year)	-0.008 ± 0.004	-0.015	-	-0.001
Temporal inferior	Baseline β_0	0.504 ± 0.024	0.457	-	0.552
	Time β_1 (year)	-0.009 ± 0.003	-0.015	-	-0.003

Supplementary Table 2 Linear mixed model in Nakanishi map

Values		Mean ± SD	95% CI		
Geometrical mean (conventional threshold sensitibities), linear model					
Superior	Baseline β_0	23.173 ± 0.678	21.844	-	24.503
	Time β_1 (year)	-0.372 ± 0.054	-0.478	-	-0.267
Temporal superior	Baseline β_0	20.481 ± 0.797	18.919	-	22.043
	Time β_1 (year)	-0.443 ± 0.081	-0.602	-	-0.285
Temporal inferior	Baseline β_0	24.129 ± 0.624	22.907	-	25.352
	Time β_1 (year)	-0.400 ± 0.068	-0.534	-	-0.267
Inferior	Baseline β_0	26.623 ± 0.395	25.848	-	27.398
	Time β_1 (year)	-0.262 ± 0.043	-0.348	-	-0.177
Arithmetic mean (anti-logged threshold sensitibities), linear model					
Superior	Baseline β_0	25.640 ± 0.387	24.881	-	26.399
	Time β_1 (year)	-0.314 ± 0.048	-0.407	-	-0.220
Temporal superior	Baseline β_0	24.431 ± 0.646	23.165	-	25.697
	Time β_1 (year)	-0.341 ± 0.065	-0.469	-	-0.214
Temporal inferior	Baseline β_0	27.455 ± 0.404	26.663	-	28.248
	Time β_1 (year)	-0.262 ± 0.043	-0.347	-	-0.178
Inferior	Baseline β_0	28.109 ± 0.212	27.693	-	28.525
	Time β_1 (year)	-0.155 ± 0.031	-0.215	-	-0.094
Geometrical mean (conventional threshold sensitibities), nonlinear model					
Superior	Baseline β_0	434.676 ± 31.641	372.661	-	496.691
	Time β_1 (year)	-22.169 ± 3.011	-28.071	-	-16.267
Temporal superior	Baseline β_0	379.072 ± 40.850	299.007	-	459.137
	Time β_1 (year)	-17.884 ± 3.242	-24.238	-	-11.530
Temporal inferior	Baseline β_0	519.193 ± 40.094	440.610	-	597.775
	Time β_1 (year)	-15.846 ± 3.987	-23.662	-	-8.031
Inferior	Baseline β_0	616.023 ± 31.543	554.200	-	677.846
	Time β_1 (year)	-17.657 ± 4.342	-26.166	-	-9.147
Arithmetic mean (anti-logged threshold sensitibities), nonlinear model					
Superior	Baseline β_0	513.151 ± 31.086	452.224	-	574.079
	Time β_1 (year)	-22.013 ± 2.975	-27.845	-	-16.182
Temporal superior	Baseline β_0	524.060 ± 39.793	446.067	-	602.053
	Time β_1 (year)	-25.019 ± 3.298	-31.482	-	-18.556
Temporal inferior	Baseline β_0	733.704 ± 36.167	662.818	-	804.589
	Time β_1 (year)	-21.765 ± 4.135	-29.870	-	-13.660

Inferior	Baseline β_0	733.359 ± 29.305	675.921	-	790.796
	Time β_1 (year)	-17.260 ± 4.589	-26.255	-	-8.266
Geometrical mean (conventional total deviation), linear model					
Superior	Baseline β_0	-5.996 ± 0.702	-7.371	-	-4.620
	Time β_1 (year)	-0.314 ± 0.056	-0.423	-	-0.205
Temporal superior	Baseline β_0	-10.692 ± 0.840	-12.338	-	-9.046
	Time β_1 (year)	-0.399 ± 0.085	-0.566	-	-0.232
Temporal inferior	Baseline β_0	-7.175 ± 0.632	-8.414	-	-5.936
	Time β_1 (year)	-0.355 ± 0.071	-0.494	-	-0.217
Inferior	Baseline β_0	-3.798 ± 0.390	-4.562	-	-3.033
	Time β_1 (year)	-0.218 ± 0.044	-0.305	-	-0.131
Arithmetic mean (anti-logged total deviation), linear model					
Superior	Baseline β_0	-3.410 ± 0.380	-4.155	-	-2.664
	Time β_1 (year)	-0.241 ± 0.048	-0.334	-	-0.147
Temporal superior	Baseline β_0	-7.021 ± 0.659	-8.313	-	-5.729
	Time β_1 (year)	-0.290 ± 0.065	-0.418	-	-0.161
Temporal inferior	Baseline β_0	-4.297 ± 0.398	-5.077	-	-3.518
	Time β_1 (year)	-0.203 ± 0.042	-0.286	-	-0.120
Inferior	Baseline β_0	-2.282 ± 0.194	-2.662	-	-1.902
	Time β_1 (year)	-0.095 ± 0.030	-0.153	-	-0.037
Geometrical mean (conventional total deviation), nonlinear model					
Superior	Baseline β_0	0.535 ± 0.038	0.460	-	0.609
	Time β_1 (year)	-0.020 ± 0.004	-0.028	-	-0.013
Temporal superior	Baseline β_0	0.308 ± 0.033	0.244	-	0.373
	Time β_1 (year)	-0.010 ± 0.003	-0.015	-	-0.005
Temporal inferior	Baseline β_0	0.385 ± 0.028	0.330	-	0.440
	Time β_1 (year)	-0.008 ± 0.003	-0.014	-	-0.001
Inferior	Baseline β_0	0.553 ± 0.026	0.501	-	0.605
	Time β_1 (year)	-0.011 ± 0.004	-0.019	-	-0.003
Arithmetic mean (anti-logged total deviation), nonlinear model					
Superior	Baseline β_0	0.632 ± 0.038	0.559	-	0.706
	Time β_1 (year)	-0.019 ± 0.004	-0.027	-	-0.012
Temporal superior	Baseline β_0	0.390 ± 0.031	0.329	-	0.451
	Time β_1 (year)	-0.014 ± 0.003	-0.019	-	-0.009
Temporal inferior	Baseline β_0	0.493 ± 0.024	0.445	-	0.540
	Time β_1 (year)	-0.009 ± 0.003	-0.015	-	-0.003

Inferior	Baseline β_0	0.660 ± 0.024	0.612 - 0.708
	Time β_1 (year)	-0.010 ± 0.004	-0.018 - -0.001

Supplementary Table 3 Linear mixed model of Spectralis cpRNFL thickness

Value		Mean ± SD	95% CI
Garway-Heath map, linear model			
Temporal superior	Baseline β_0	85.901 ± 3.040	79.943 - 91.858
	Time β_1 (year)	-1.600 ± 0.160	-1.913 - -1.287
Temporal	Baseline β_0	62.627 ± 1.791	59.117 - 66.138
	Time β_1 (year)	-0.903 ± 0.116	-1.129 - -0.676
Temporal inferior	Baseline β_0	77.167 ± 2.670	71.933 - 82.401
	Time β_1 (year)	-1.664 ± 0.189	-2.034 - -1.294
Garway-Heath map, nonlinear model			
Temporal superior	Baseline β_0	4.379 ± 0.038	4.306 - 4.453
	Time β_1 (year)	-0.021 ± 0.002	-0.026 - -0.016
Temporal	Baseline β_0	4.090 ± 0.030	4.030 - 4.149
	Time β_1 (year)	-0.015 ± 0.002	-0.018 - -0.012
Temporal inferior	Baseline β_0	4.273 ± 0.036	4.202 - 4.343
	Time β_1 (year)	-0.025 ± 0.003	-0.031 - -0.019
Nakanishi map, linear model			
Superior	Baseline β_0	82.164 ± 1.829	78.546 - 85.783
	Time β_1 (year)	-0.936 ± 0.109	-1.153 - -0.719
Temporal superior	Baseline β_0	84.782 ± 2.552	79.781 - 89.783
	Time β_1 (year)	-1.392 ± 0.135	-1.658 - -1.127
Temporal inferior	Baseline β_0	70.761 ± 2.840	65.193 - 76.328
	Time β_1 (year)	-1.608 ± 0.160	-1.922 - -1.295
Inferior	Baseline β_0	76.298 ± 2.042	72.296 - 80.301
	Time β_1 (year)	-1.031 ± 0.147	-1.319 - -0.743
Nakanishi map, nonlinear model			
Superior	Baseline β_0	4.371 ± 0.025	4.321 - 4.421
	Time β_1 (year)	-0.012 ± 0.001	-0.015 - -0.010
Temporal superior	Baseline β_0	4.386 ± 0.032	4.322 - 4.449
	Time β_1 (year)	-0.018 ± 0.002	-0.022 - -0.014
Temporal inferior	Baseline β_0	4.164 ± 0.041	4.083 - 4.245
	Time β_1 (year)	-0.025 ± 0.003	-0.030 - -0.020
Inferior	Baseline β_0	4.285 ± 0.030	4.225 - 4.344
	Time β_1 (year)	-0.017 ± 0.003	-0.023 - -0.011
